# The complete chloroplast genome sequence of *Acanthopanax trifoliatus (Linn.) Merr.*

**DOI:** 10.1080/23802359.2020.1823900

**Published:** 2020-09-23

**Authors:** Jingyu Li, Fang Zhou, Yu Mei, Yan Gu, Shiqang Xu, Shike Cai, Mingyang Sun, Jihua Wang

**Affiliations:** Key Laboratory of Crops Genetic Improvement of Guangdong, Crops Research Institute, Guangdong Academy of Agricultural Sciences, Guangzhou, China

**Keywords:** *Acanthopanax trifoliatus (Linn.) Merr.*, chloroplast genome, medical plant

## Abstract

*Acanthopanax trifoliatus (Linn.) Merr.* is an edible vegetables and medicinal plant from Asian countries. In this study, the complete chloroplast genome of *A. trifoliatus* was assembled and annotated by high-throughput sequencing. The total chloroplast genome size of *A. trifoliatus* was 156,716 bp, containing a large single-copy (LSC) region of 86,672 bp, a small single-copy (SSC) region of 18,174 bp, and a pair of inverted repeat regions of 25,935 bp. A total of 134 genes were predicted in the chloroplast genome of *A. trifoliatus*, including 89 protein-coding genes, 37 tRNA genes, and 8 rRNA genes. Phylogenetic analysis showed that *A. trifoliatus* was closely related to *Eleutherococcus gracilistylus*.

*Acanthopanax trifoliatus* is an edible medicinal plant belonging to the *Acanthopanax* genus in the Araliaceae family. It is commonly cultivated in China, India, Thailand, Vietnam, and the Philippines. Leaves of *A. trifoliatus* are popularly consumed as vegetables and herbal tea in southern China (Chen et al. 2019). Traditionally, *A. trifoliatus* has also been used as folk medicine to treat various diseases, including lung hemorrhages, bruises, ulcers, and partial (Sithisarn et al. 2009). Pharmacological studies also reported that *A. trifoliatus* extracts showed numerous biological activities, including antioxidative, anti-inflammatory, anti-cancer, and neuroprotective effects (Chen et al. 2019). The sequencing of the chloroplast genome of medicinal plants will promote in-depth research on the chloroplast genome of medicinal plants and the development of related industries. In this study, we assembled the complete chloroplast genome of *A. trifoliatus* to provide genomic and genetic sources for further research.

The fresh leaves of *A. trifoliatus* were collected from Guangdong Academy of Agricultural Sciences (Guangzhou, China, N23.1459, E113.3498). The samples were frozen in liquid nitrogen and stored at −80 °C refrigerator until use at Key Laboratory for Crops Genetic Improvement of Guangdong in Guangdong Academy of Agricultural Sciences (specimen code LC2020). Total genomic DNA was extracted using plant genomic DNA kit (Omega, Norcross, GA) and then constructed DNA library with average insert size 350-bp using VAHTS Universal DNA Library Prep Kit (Vazyme ND606-01, Nanjing, China). Finally, the library was sequenced on the Novaseq platform (Illumina, San Diego, CA) following as recommended by the manufacturer. The sequence was assembled by the GetOrganelle (Jin et al. 2019) and annotated with the Geseq (Tillich et al. 2017) using the cp annotation of *Fatsia japonica* (NC_027685) as a reference. Then, the chloroplast genome sequence was submitted to GenBank with the accession number MT754220.

The total genome size of *A. trifoliatus* was 1,56,716 bp, containing an large single-copy (LSC) region of 86,672 bp, a small single-copy (SSC) region of 18,174 bp, and a pair of IRs region of 25,935 bp. A total of 134 genes were predicted, including 89 protein-coding genes, 37 tRNA genes, and eight rRNA genes, and the GC content was 38.02%.

Phylogenetic analysis suggested that *A. trifoliatus* was closely clustered with *Eleutherococcus gracilistylus* (Figure 1), which was generated based on the 18 complete chloroplast genomes using maximum likelihood method by RaxML software (Stamatakis 2014) using the GTRGAMMA nucleotide substitution model. The chloroplast genome sequence of *A. trifoliatus* will provide useful genetic information for further study on molecular breeding and genetic engineering.

**Figure 1.**
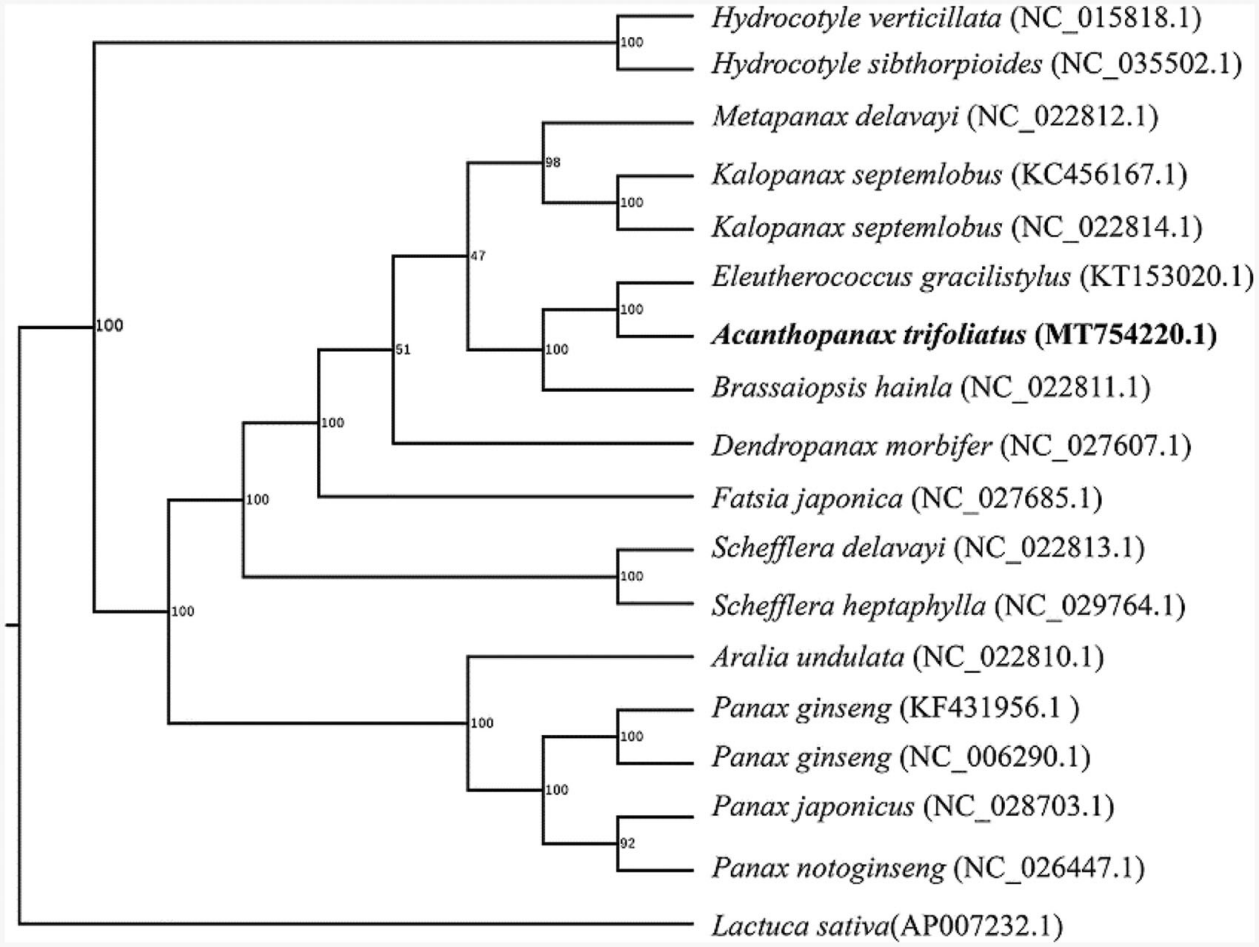
The phylogenetic tree of the *A. trifoliatus (Linn.) Merr.* and other species based on the 18 complete chloroplast genome sequences with 1000 bootstrap replicates.

## Data Availability

The data that support the findings of this study are openly available in GenBank (https://www.ncbi.nlm.nih.gov/nuccore/ MT754220.1), and the assession number is MT754220.1.

## References

[CIT0001] Chen M, Qin Y, Ma H, Zheng X, Zhou R, Sun S, Huang Y, Duan Q, Liu W, Wu P, et al. 2019. Downregulating NF-κB signaling pathway with triterpenoids for attenuating inflammation: in vitro and in vivo studies. Food Funct. 10(8):5080–5090. 31361289 10.1039/c9fo00561g

[CIT0002] Jin JJ, Yu WB, Yang JB, Song Y, Yi TS, Li DZ. 2019. GetOrganelle: a fast and versatile toolkit for accurate de novo assembly of organelle genomes. BioRxiv. 256479. 10.1186/s13059-020-02154-5 PMC7488116 32912315

[CIT0003] Sithisarn P, Jarikasem S, Thisayakorn K. 2009. Anti-inflammatory and antioxidative effects of leaf extract from *Acanthopanax trifoliatus*. Planta Med. 75(9):891–891.

[CIT0004] Stamatakis A. 2014. RAxML version 8: a tool for phylogenetic analysis and post-analysis of large phylogenies. Bioinformatics. 30(9):1312–1313. 24451623 10.1093/bioinformatics/btu033 PMC3998144

[CIT0005] Tillich M, Lehwark P, Pellizzer T, Ulbricht-Jones ES, Fischer A, Bock R, Greiner S. 2017. GeSeq- versatile and accurate annotation of organelle genomes. Nucleic Acids Res. 45(W1):W6–W11. 28486635 10.1093/nar/gkx391 PMC5570176

